# New Host Range for *Hematodinium* in Southern Australia and Novel Tools for Sensitive Detection of Parasitic Dinoflagellates

**DOI:** 10.1371/journal.pone.0082774

**Published:** 2013-12-06

**Authors:** Sebastian G. Gornik, Andrea Cranenburgh, Ross F. Waller

**Affiliations:** 1 School of Botany, University of Melbourne, Melbourne, Victoria, Australia; 2 Department of Biochemistry, University of Cambridge, Cambridge, United Kingdom; National Science and Technology Development Agency, Thailand

## Abstract

*Hematodinium* is a parasitic dinoflagellate and emerging pathogen of crustaceans. It preferably manifests in haemolymph of marine decapod crustaceans, killing a large variety of genera with significant impacts on fisheries worldwide. There is, however, evidence that some crustacean stocks harbor high prevalence, low intensity infections that may not result in widespread host mortality and are therefore hard to detect. The most widely used methods for detection of *Hematodinium* are conventional blood smears and polymerase chain reaction (PCR) against ribosomal RNAs. Blood smears demand a trained investigator, are labor intensive and not readily scalable for high-throughput sampling. PCRs only detect parasite DNA and can also suffer from false negatives and positives. In order to develop alternative detection tools for *Hematodinium* cells in decapod crustaceans we employed an immunological approach against a newly identified, abundant dinoflagellate-specific nuclear protein—Dinoflagellate/Viral NucleoProtein (DVNP). Both immunofluorescence assay (IFA) and Western blot methods against DVNP showed high sensitivity of detection. The Western blot detects *Hematodinium* parasites to levels of 25 parasites per milliliter of crustacean haemolymph, with the potential for sample pooling and screening of large samples. Using both PCR and these new tools, we have identified *Hematodinium* cells present in three new host crab taxa, at high prevalence but with no sign of pathogenesis. This extends the known range of *Hematodinium* to southern Australia.

## Introduction

*Hematodinium* is a genus of parasitic dinoflagellates that infect the haemolymph of marine decapod crustaceans [1]. Various genotypes of *Hematodinium* occur, however, it is not known exactly how many species of *Hematodinium* exist and to date only two species of *Hematodinium* are formally described: the type species *Hematodinium perezi* found in European waters [2] and *Hematodinium australis* from Australia [3]. In advance stages of infection the parasite cells proliferate exponentially inside their host and often several millions of parasite cells can be found per milliliter of crustacean blood (haemolymph) [4]. Patent infections with *Hematodinium* are characterized by a change in the color and consistency of haemolymph and tissue effluents from translucent to milky-white and eventually custard-yellow as parasites proliferate [1]. Affected hosts are increasingly unable to circulate oxygen, become permanently stressed, lethargic and eventually suffocate [5,6]. 

During the past four decades, epizootics of *Hematodinium* were described from many decapod crustacean stocks in the USA, the UK, Europe and Australia and often cause significant economical loss to associated fisheries [1,7,8]. When the disease is at its peak, moribund, diseased animals can make up 70-100% of the catch [9]. In France *Hematodinium* epizootics have led to the closure of a principal fishery [10]. Elsewhere the parasite is considered a significant factor driving long-term stock reductions [11-13]. Extensive field surveys in some decapod stocks suggest that clear seasonal peaks with high intensity infections and disease are interspersed with periods of low infection intensity [14] during which prevalence remains high [15]. This suggests slow progression to disease in large numbers of the population. There is also evidence that some populations of crustaceans may be hosts to asymptomatic *Hematodinium* infections, and disease is only triggered by physiological or environmental stresses [16]. The seasonal nature of disease outbreaks and the influence of environmental factors raises the possibility that the host-range of *Hematodinium* may be greater than might be suggested by monitoring only for the incidence of disease.

Understanding the prevalence of *Hematodinium*, and its potential to develop into disease in both wild and commercially important crustacean stocks, requires effective detection methods. Currently, the most sensitive method for detection of *Hematodinium* is the use of polymerase chain reaction (PCR) to amplify a fragment of *Hematodinium* ribosomal DNA [15,17-19]. Recently, a nested PCR approach has been employed to improve sensitivity [16]. The alternative to PCR detection is histological staining and examination of haemolymph smears. Although *Hematodinium* has a distinctive appearance, notably a small number of condensed chromosomes filling the nucleus that can be observed with stains that react with DNA, the sensitivity of this method is dependent on: the quality of sample preparation; the density of crustacean haemocytes, that can have a similar presentation; and the experience of the microscopist [4,20,21]. These requirements limit the application of this approach.

In this study we have investigated the presence of *Hematodinium* in crustacean populations from Port Phillip Bay (Victoria), Australia. *Hematodinium* has not previously been reported from southern Australia, and using a nested PCR approach we find evidence of a high prevalence of the parasite in this new geographic region, and in at least three new crustacean hosts. Histological examination, however, indicates only very low infection levels, and we found no evidence of the severe disease often found elsewhere. 

In pursuit of alternative tools to observe lowly infected hosts, and to confirm the presence of viable parasites, we have exploited presence of a highly abundant DNA-binding, nuclear protein that has recently been identified from *Hematodinium* [22]. Dinoflagellate viral nucleoproteins (DNVPs) are unique to dinoflagellates and are highly antigenic proteins allowing ready detection by immunological approaches. We have explored the use of DVNP antibodies for detection of *Hematodinium* cells by both immunofluorescence assay (IFA) and Western blots, and identify this as a potent new strategy in detecting and/or corroborating presence of this parasite in decapod host samples.

## Materials and Methods

### Sources of *Hematodinium* and live crustaceans

*Ovalipes australiensis* (sand crabs), *Leptomithrax gaimardii* (giant spider crabs) and *Plagusia chabrus* (red bait crab) were caught in lobster traps (pots) baited with squid and pilchard and submerged overnight in Port Phillip Bay monthly from February to June 2011. Additionally, *Scylla serrata* (mud crabs) were obtained live from local fish markets to harvest fresh haemolymph during Western blot development. Permits for trapping, sampling and maintenance of wild animals were obtained from the Department of Primary Industries (Victoria, Australia; permit number RP1017). *Hematodinium* sp. cells (ex Norway lobster *Nephrops norvegicus*) were maintained in the dark at 10°C as described in Gornik et al. (2012) [22].

### Maintenance and observation of captive crabs

*Leptomithrax gaimardii* caught in February 2011 were maintained in flow-through seawater tanks at ambient temperatures at the Victorian Marine Science Consortium research laboratories in Queenscliffe (Victoria, Australia). 

### Haemolymph sampling, histology and DNA extraction

Haemolymph was visually inspected for color and consistency and tested for *Hematodinium* by PCR as described below and by inspection of haemolymph smears. Approx. 100 µl of haemolymph were withdrawn from live crabs using sterile needles and syringes inserted between the walking legs (pereopods) following surface disinfection with 75% ethanol. Haemolymph smears were prepared by smearing 50 µL of haemolymph on a glass slide. Slides were immediately fixed and stained using a Diff-quick^®^ staining kit (Polysciences) according to the manufacturers instructions and mounted under a coverslip for microscopic examination with a 100X oil immersion lens. The remaining 50µl of haemolymph were immediately subjected to DNA extraction. 50 µL of haemolymph was combined with 150 µl of DNAzol (Invitrogen) and 150 µl of chloroform, vortexed, incubated for 5 minutes at room temperature and centrifuged for 10 minutes at 12,000 g to remove debris. The aqueous phase of the supernatant was removed and combined with 120 µl of ethanol, centrifuged at 5,000 g for 4 minutes, and the resulting DNA pellets were washed twice in 75% ethanol, air-dried and solubilized in 30 µL of sterile MilliQ water.

### Nested PCR, cloning and sequencing

Routine screening for *Hematodinium* was carried out using a nested PCR to amplify a 403 bp fragment (excluding primers sequence) of the *Hematodinium* 18S ribosomal DNA gene (18S rDNA). For the first round of amplification, 20 µL PCR reaction mixtures were prepared using: 1 µL of DNA; 0.2 µL iTaq DNA polymerase [10 U/µL] (Scientifix); 2 µL iTaq buffer; 2 μL deoxynucleoside triphosphates [dNTPs; 2.5 mM]; 1.5 µL forward primer F1 [CGAACCAAGCTCTGCTTGGCC; 10 µM]; 1.5 µL reverse primer R1 [GTAGGTGAACCTGCGGAAGGATC; 10 µM]; 11.8 µL MilliQ water. PCR conditions were: initial denaturation at 94°C for 5 minutes followed by 35 cycles of 94°C for 15 seconds, 55°C for 15 seconds 72°C for 90 second with a final extension for 72°C for 2 minutes following the last cycle. Often template DNA had to be diluted 1 to 10 or 1 to 100 times for the first round of PCR to achieve amplification in the second round. 1 µL of product from the first round of amplification was combined with a PCR reaction mix containing 0.2 µL iTaq DNA polymerase [10 U/µL]; 2 µL iTaq buffer; 2 µL dNTPs [2.5mM]; 1.5 µL forward primer F2 [CTGCTTGAAGCGATCGGTGC; 10 µM]; 1.5 µL reverse primer R2 [CATAAGGTGCTGAAGGTGTCGTC; 10 µM]; 11.8 µL MilliQ water. Second round PCR conditions: 94°C for 5 minutes; 35 cycles of 94°C for 15 seconds, 55°C for 15 seconds, 72°C for 45 seconds; and 72°C for 2 minutes. PCR amplicons were excised from agarose gels, purified using an Agarose GelExtract Mini (5Prime), ligated into the pGEM-T easy plasmid vector (Promega), transformed into *E. coli* TOP10 cells, and the inserts bidirectionally sequenced. Host-specific unique *Hematodinium* 18S rDNA sequences were deposited at GenBank (Accession No. KF460025- KF460027).

### Immuno-fluoresecence assay (IFA)

The authors have previously prepared an anti- DVNP antibody (see Gornik et al. (2012) [22]), which is available upon request. Haemolymph withdrawn from live crabs was immediately mixed 1:1 with crustacean anticoagulant [0.45 M NaCl; 0.1M glucose; 30 mM trisodium citrate; 26mM citric acid; 10 mM EDTA]. Cells were separated from serum by centrifugation for 5 minutes at 2,000 rpm. Cells were fixed for 15 minutes in crustacean anticoagulant containing 4% paraformaldehyde, resuspended in 0.1M glycine in Phosphate-buffered saline (PBS) to quench the fixation and stored at 4°C. Immediately before use, fixed cells were permeabilised by incubating for 10 minutes in PBS containing 0.1% (v/v) Tween 20. Ethanol-preserved haemolymph from heavily infected *Callinectes sapidus* from Cheasapeake Bay (USA) were prepared for IFA as follows: 20 µL of preserved haemolymph was rehydrated by slowly adding 20 µL of MilliQ water followed by 40 µL of PBS. Cells were separated by centrifugation for 30 seconds at 2000 rpm and washed 3 times in PBS.

Prepared cells were blocked for 20 minutes in PBS containing 2% (w/v) bovine serum albumin (BSA) then incubated for 30 minutes in the primary antibody solution [0.5% BSA/PBS containing a 1:750 dilution of the polyclonal anti-DVNP rabbit antiserum as described by Gornik et al. (2012) [22]. Cells were washed 3 times for 5 minutes in PBS and then incubated in the dark for 30 minutes in the presence of secondary antibody [0.5% BSA/PBS containing a 1:1000 dilution of Alexa Fluor 488 goat-anti-rabbit antibodies (Invitrogen)]. Cells were washed again with the final wash containing 1 µg ml^-1^ propidium iodide as a DNA counterstain. Cells were mounted in 10 µl of FluoroGel (ProSciTech) for examination with a confocal laser-scanning microscope.

### Sample preparation for Western blot

Cells were extracted from *Hematodinium* sp. ex *Nephrops norvegicus* culture by centrifuging at 800 g at 10°C for 5 minutes. Cells were resuspended in *Nephrops* saline and the number of parasites per microliter was counted using an improved Neubauer counting chamber. The cell suspension was serially diluted with *Nephrops* saline to achieve a gradient of cell densities. Simulated infected samples were prepared by combining haemolymph or plasma-free cells from *S. serrata* (pelleted at 2000 rpm, 2-3 minutes) with dilutions of *Hematodinium* cells to make up the desired number of parasites ml^-1^. Samples were mixed with 6x SDS loading buffer [375 mM Tris-HCl pH 6.8, 6% (w/v) SDS, 48% (v/v) glycerol, 9% (v/v) 2-Mercaptoethanol, and 0.03% (w/v) bromophenol blue], heated for 10 minutes to 70°C. 

Acid-soluble nuclear proteins were extracted from simulated samples and *Hematodinium* PCR-negative haemolymph as described in Shechter et al. (2007) [23]. Briefly, 100 µL of haemolymph (with or without added parasites) were mixed 1:1 with crustacean anticoagulant. Cells were pelleted (5 minutes, 2000 rpm), resuspended in a modified hypotonic lysis buffer [10 mM Tris–Cl pH 8.0; 1 mM KCl; 1.5 mM MgCl_2_; 1 mM DTT; 5mM EDTA; complete™ protease inhibitor cocktail (Roche)] and incubated with constant rotation for 30 minutes at 4°C to lyse cell membranes. Nuclei were pelleted for 10 minutes at 10,000 g at 4°C, resuspended in 0.4N H_2_SO_4_ and incubated overnight at 4°C with constant rotation. Insoluble material was pelleted at 16,000 g at 4°C for 10 minutes, proteins were precipitated from the supernatant by trichloroacetic acid (TCA) precipitation, washed twice in ice-cold acetone, solubilized in 60 µl of MilliQ water and prepared for SDS-PAGE with 6x SDS loading buffer at 70°C for 10 minutes.

### Western blot

If not otherwise stated, per sample 20-25 µg of protein were size separated on NuPAGE 4-12% polyacrylamide gels (Invitrogen) via SDS-PAGE and transferred onto nitrocellulose membrane [GE Nitropure, 0.22µm] using standard protocols. Membranes were blocked for 1 hour in PBS containing 5% (w/v) skim milk powder. Successively, the membrane was incubated for 1 hour with the primary antibody [anti-DVNP rabbit antiserum diluted 1:20,000 in 1% skim milk/PBS] and incubated an additional 30 minutes with secondary antibody [goat anti-rabbit immunoglobin G (IgG) conjugated with horseradish peroxidase (HRP) (BioRad), diluted 1:10,000 in 1% skim milk/PBS]. To remove any unbound antibodies, membranes were washed 3 times in 0.1% Tween 20/PBS after the application of the primary antibody and again after the application of the secondary. Western blot signal was visualized using ECL Prime Western Blotting Detection Reagent (GE Amersham) and Hyperfilm ECL (GE Amersham).

## Results

### *Hematodinium* occurs in several decapod crustacean species in Port Philip Bay

**Figure 1:**
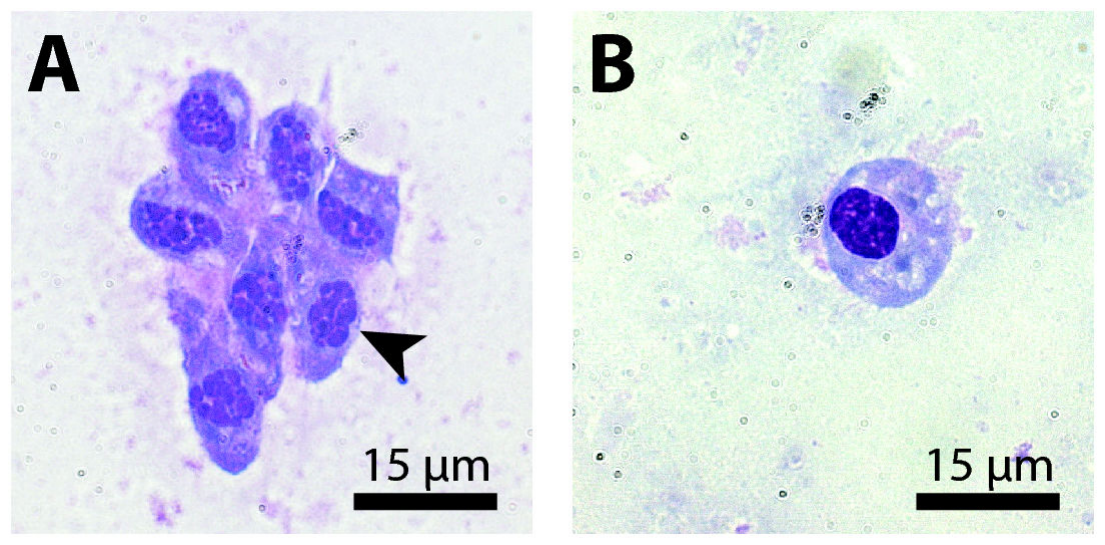
Diff-quick^®^-stained haemolymph smears of *O. australiensis* from Port Philip Bay. (A) Aggregation of *Hematodinium* sp. cells displaying a *Hematodinium*-typical nuclear appearance (black arrow) (B) *O. australiensis* haemocyte. Note the different appearance of the chromatin in comparison to the parasite cells. Scale-bar: 15 µm.

Between June 2010 and February 2011 crustacean haemolymph samples from Port Philip Bay were tested for the presence of *Hematodinium* using nested PCR and Diff-quick^®^-stained haemolymph smears. Of 214 animals 164 (77%) tested positive for the parasite by PCR (Table 1). A random subset of the several PCR amplicons was sequenced to verify identity with *Hematodinium* DNA. Some minor sequence variability was observed, apparently indicating genetic novelty in these Australian samples (see Figure S1). All of these crustaceans represent newly identified *Hematodinium* hosts, and include: the sand crab *Ovalipes australiensis*; the spider crab *Leptomithrax gaimardii*; and the red bait crab *Plagusia chabrus*. Despite the high prevalence *Hematodinium*, only one of the PCR-positive crabs was observed as positive for parasites by microscopic examination following Diff-quick^®^-staining. In this individual parasite load was very low, but the distinctive presentation of *Hematodinium* nuclei was observable (Figure 1). 

**Table 1:** *Hematodinium* PCR-positive host species detected from Port Philip Bay (June 2010 to February 2011).

Host Species	Sample size	No. PCR-positive [percent]
*Leptomithrax gaimardii*	121	105 [87%]
*Plagusia chabrus*	49	26 [53%]
*Ovalipes australiensis*	44	33 [75%]
**Total**	**214**	**164 [77%]**

To investigate whether PCR-positive crabs would develop patent *Hematodinium* infections over time, six PCR-positive spider crabs (*L. gaimardii*) were kept in captivity between February 2011 and February 2012 and screened by PCR each month. Interestingly, during captivity some animals alternated between positive and negative PCR results indicating either fluctuating levels of parasites in their haemolymph (potentially due to sessile life stages that do not circulate), or the detection method being close to its sensitivity threshold. In all cases PCR amplicons were cloned and sequenced and found to match the 403 bp target sequence of *Hematodinium* 18S rDNA. Throughout the study period *Hematodinium* cells were not detected in haemolymph smears of the 6 PCR-positive spider crabs when stained with Diff-quick^®^ indicating that infection intensity remained very low.

### DVNP immuno fluorescence assays (IFAs) clearly differentiates parasites from host cells

**Figure 2:**
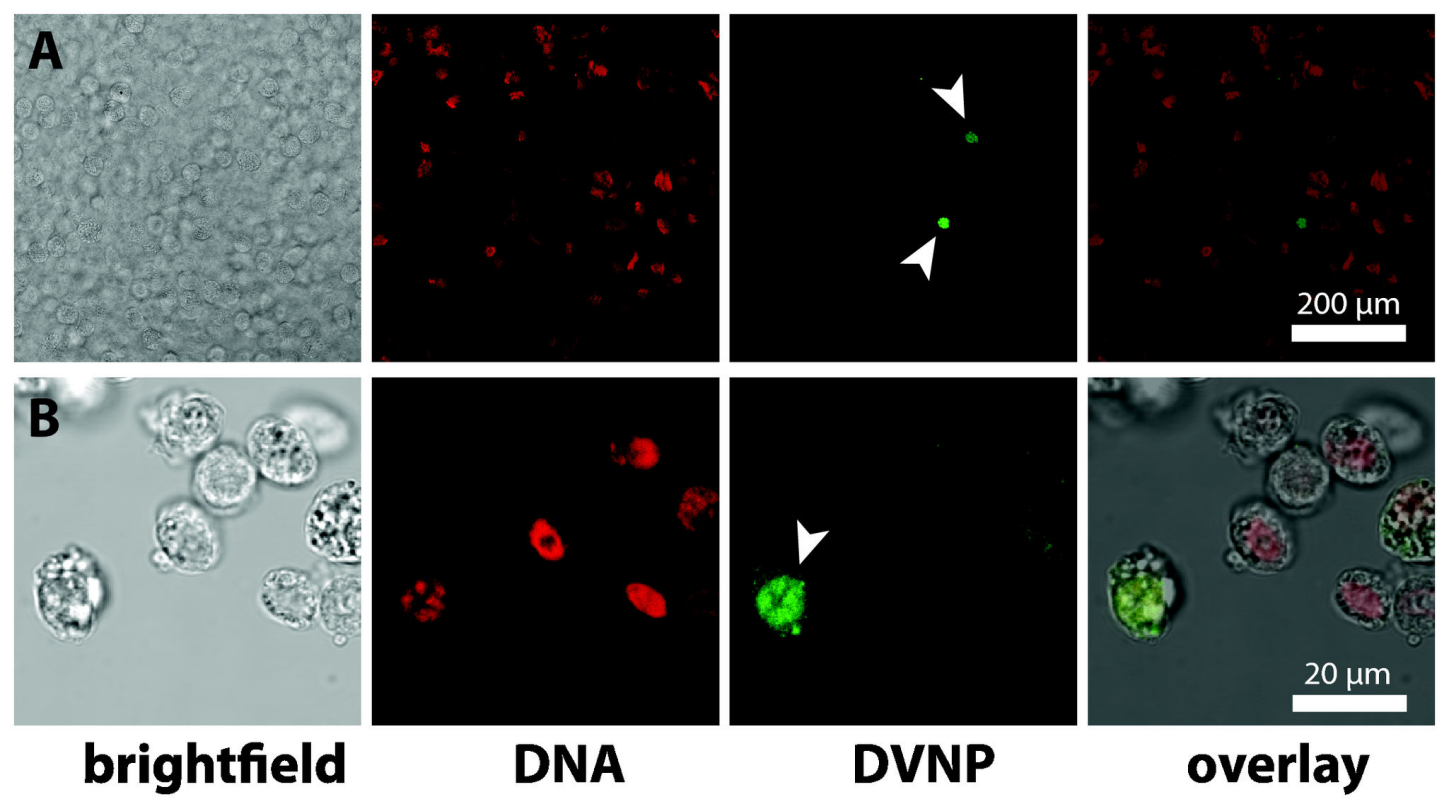
DVNP IFAs of haemolymph parasite mixes. The DNA is shown in red and anti-DVNP is shown in green. (A) Haemolymph smear of artificially infected sand crab *O. australiensis* showing two DVNP-positive cells (white arrowsheads) within a large number of haemocytes. (B) Haemolymph smear of artificially infected spider crabs *L. gaimardii* showing one DVNP-positive *Hematodinium* cell amongst haemocytes.

**Figure 3:**
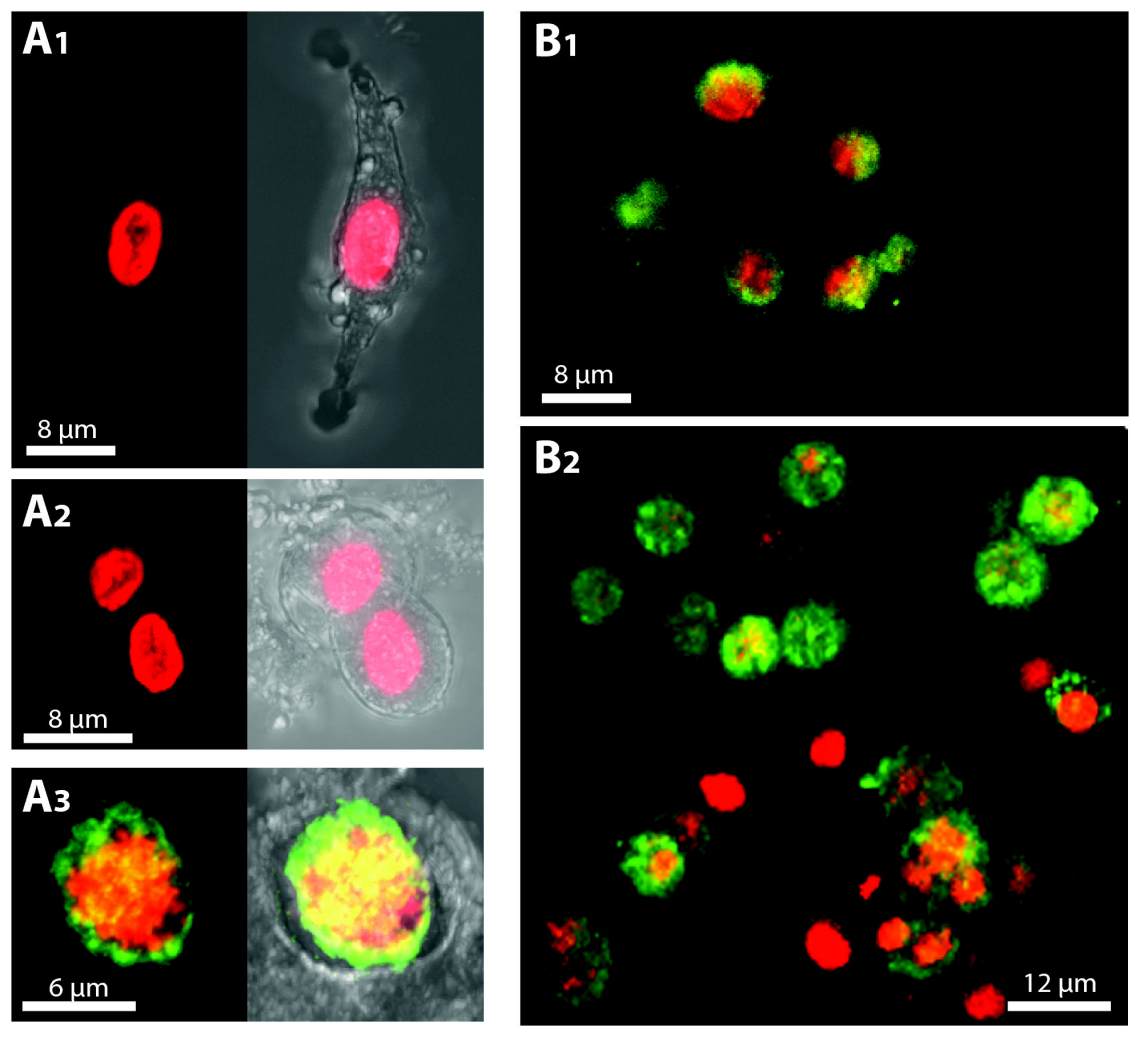
DVNP IFAs of ethanol-fixed haemolymph samples obtained from heavily infected blue swimmer crabs *Calinectes sapidus* from Chesapeake Bay (USA). DNA is shown in red and anti-DVNP is shown in green. (A1) IFA (left) and brightfield overlay (right) of a DVNP-negative *C. sapidus* hyalinocyte blood cell. (A2) IFA (left) and brightfield overlay (right) of a DVNP-negative *C. sapidus* granulocyte blood cell. (A3) IFA (left) and brightfield overlay (right) of a DVNP-positive *Hematodinium* cell. (B1) *Hematodinium* cells are strongly labeled around the edge of their nuclei while the DNA stain concentrates in the center of the nuclei. (B2) DVNP-labeling is absent in *C. sapidus* cells (red nuclei) and prominent in *Hematodinium* cells (green-yellow nuclei).

**Figure 4:**
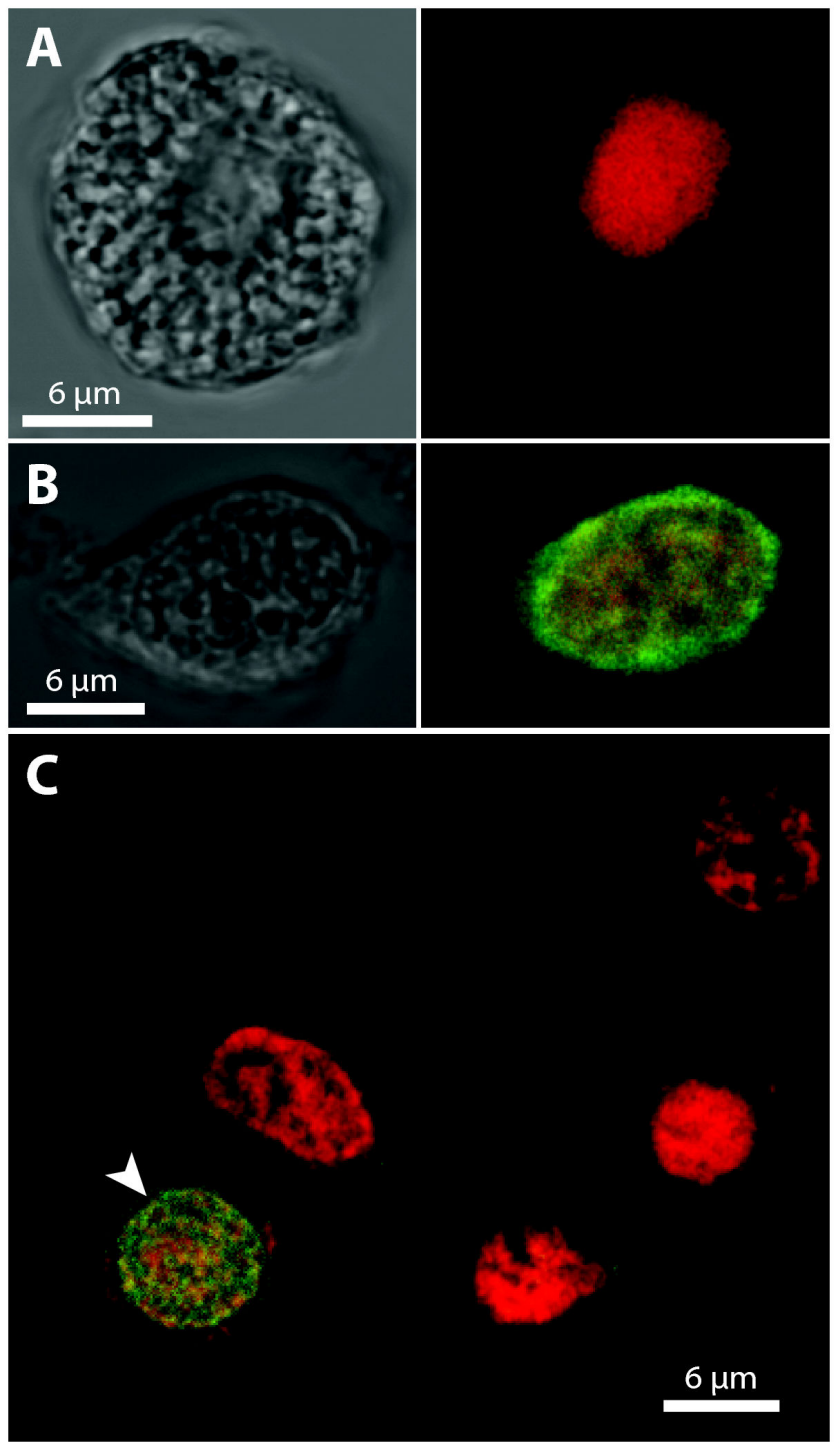
DVNP IFAs of fixed haemolymph from PCR-positive *L. gaimardii* from Port Phillip Bay, Australia. (A) Brightfield image (left) and IFA (right) of a DVNP-negative *L. gaimardii* haemocytes. (B) Brightfield image (left) and IFA (right) of a DVNP-positive *Hematodinium* cell. (C) IFA image of one DVNP-positive *Hematodinium* cell (white arrowhead) amongst *L. gaimardii* haemocytes.

DVNP IFA labeling of *Hematodinium* and host cells was initially assessed using cultured *Hematodinium* cells and haemolymph from PCR-negative locally caught sand crabs (*O. australiensis*) and spider crabs (*L. gaimardii*). The haemolymph was mixed with cultured *Hematodinium* cells to test if the antibody could distinguish host from parasite cells. The DVNP antibody consistently labeled the nuclei of *Hematodinium* sp. but labeling was absent from nuclei of crustacean haemocyotes (Figure 2). Thus, the IFA provided unequivocal means of differentiating parasites from host cells in this artificial scenario, even in high cell density samples where cells are crowded and potentially overlayed (Figure 2A). To test if the IFA could also detect *Hematodinium* cells in field samples we first examined heavily infected blue swimmer crabs *Calinectes sapidus* from Chesapeake Bay (USA) that had been previously preserved in ethanol. DVNP-label was absent in cells that morphologically matched *C. sapidus* haemocytes (Figure 3 A1 and A2). *Hematodinium* cells, on the other hand, were strongly labeled around the edge of their nuclei with DNA stain signal concentrated in the center (Figure 3 A3, B1 and B2). A control sample of cultured *Hematodinium* cells fixed overnight in 95% ethanol showed a similar separation of the DVNP-label and DNA-stain, indicating that this signal separation was an artifact of the ethanol fixation/rehydration process (data not shown). We also investigated whether *Hematodinium* could be detected by anti-DVNP IFA in freshly fixed haemolymph of PCR-positive *L. gaimardii* from Port Phillip Bay that were maintained in captivity. DVNP-positive cells were found in all samples (Figure 4) although only after examining several fields of view, consistent with low parasite abundance. These cells displayed morphology consistent with that described for *Hematodinium* trophonts found in other Australian hosts [3]. Accurate assessment of infection intensity in *L. gaimardii* from Port Phillip Bay was not possible due to the rarity of the parasites in these samples, however the clear distinction of parasite nuclei attests to the sensitivity of this approach.

### DVNP Western blot detects *Hematodinium* cells with a sensitivity of 25 cells/ml of haemolymph

The suitability of the DVNP antibody for the detection of *Hematodinium* cells in haemolymph extracts from decapod crustaceans using a Western blot approach was tested also. Initially, Western blots were conducted on total proteins extracted from *Hematodinium* cultures either with or without addition of proteins extracted from either whole mud crabs (*Scylla serrata*) haemolymph or *S. serrata* haemocytes separated by centrifugation from the plasma. While a strong signal was produced by a positive control of 1 µg of recombinant DVNP, the pure *Hematodinium* lysate representing the equivalent of 1,200 cells produced only a weak signal (Figure S2), and no DVNP was detected from parasite samples mixed with crab haemolymph samples (equivalent to 1500, 300 and 30 parasites ml^-1^ of haemolymph). Coomassie staining of the protein samples indicated abundant protein in all haemolyph samples (not shown) that might be an impediment to DVNP detection sensitivity. Fortunately DVNPs are highly basic, lysine rich proteins (pI ranging from 10.5-12). This enables their selective solubilization by acid extraction [22]. 

**Figure 5:**
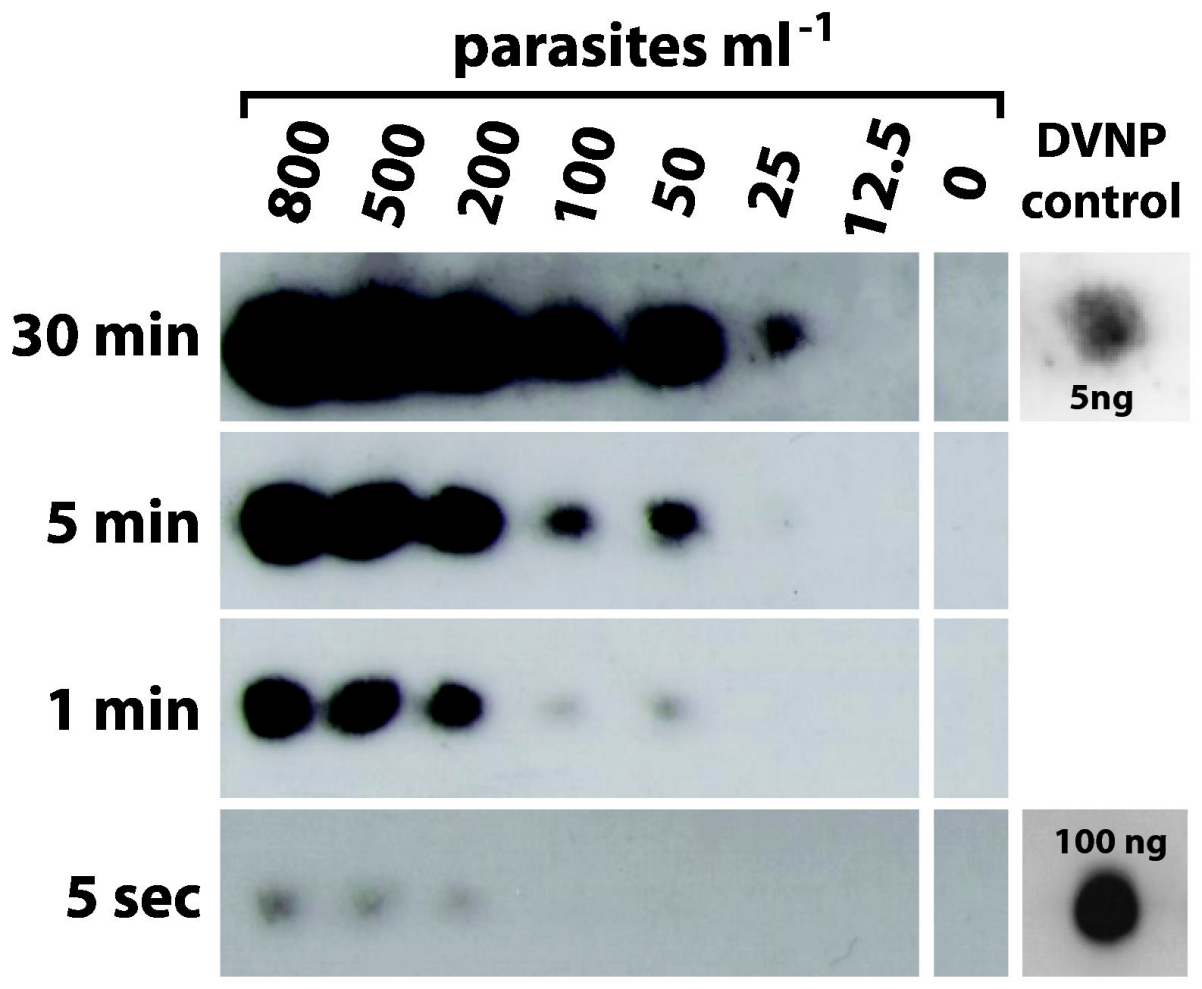
Western blot detection of DVNP in haemolymph-parasite mixes after target enrichment using acid extraction. Samples containing 800, 500, 200, 100, 50, 25, 12.5, 5, 3 and 0 parasites ml^-1^ of haemolymph.

To enrich for the target protein in the samples acid-soluble protein extracts were prepared. Samples containing 800, 500, 200, 100, 50, 25, 12.5, 5, 3 and 0 parasites ml^-1^ of haemolymph were tested. Reactive bands of size consistent with DVNP were detected to a sensitivity of 25 parasites ml^-1^ of haemolymph (Figure 5). This represents a significant increase from the previously observed 3,000 parasites ml^-1^ detection limit in the non-enriched samples. Recombinant DVNP was used as a control and detected to 5 ng. No significant cross-reactivity with non-target proteins was detected while Coomassie-staining of the size-separated proteins revealed that the acid-soluble protein extracts still contained a wide range of protein species (not shown). Only with very long overexposures was some cross-reactivity with high molecular weight proteins was evident, but these are easily distinguished from the DVNP size range of 20-25 kDa. 

## Discussion

The host range of *Hematodinium* is very broad and worldwide many species of crustaceans are infected by this parasite [1]. Over the last 2 decades the list of identified hosts species has been extended dramatically [2,8]. As *Hematodinium* is identified in more and more regions and host species, it becomes apparent that the parasite does not always cause fast progressing infections as previously thought. Some hosts, in fact, apparently carry slow progressing, latent infections, that only rarely lead to disease, presumably upon certain environmental or physiological stresses [16]. Such types of low-intensity infections are hard to detect, especially using ‘classical’ histological methods. In this study, using a nested PCR approach, we have found evidence of *Hematodinium* at a new geographic location of southern Australia (Port Phillip Bay, Victoria, Australia), and associated with three new crab hosts. In all samples only low levels of infections were found, and maintenance of infected animals in captivity over 12 months did not lead to progression of the infection. This suggests that these hosts might tolerate and control *Hematodinium* infections, although it is possible that with the right triggers disease might develop. A similar early detection of a new host population was initially limited to nested PCR [16], and this study and ours highlight the need for alternative detection strategies to corroborate PCR results. Typically histology via blood smears is used. When we applied this standard method to the PCR-positive samples from Port Phillip Bay, however, we observed *Hematodinium* cells in only one of the positive animals, and only at very low parasite abundance. 

In response to the need for alternative, sensitive detection methods, we have developed two additional diagnostic methods for the detection of *Hematodinium* cells within their decapod hosts based on antibodies. We have used antibodies against an abundant, highly antigenic protein, the nuclear protein DVNP that is unique to dinoflagellates [22]. DVNP is strongly conserved amongst dinoflagellates, so these detection tests are not in themselves specific for *Hematodinium* amongst other parasitic dinoflagellates. However, while other genera of dinoflagellates are known to parasitize either external or internal compartments of various crustaceans, *Hematodinium* is currently the only known dinoflagellate parasite of decapod crustaceans, where it is also most commercially relevant [24]. Therefore these sensitive tests that we have developed will have broad utility in known host systems. They will have utility also in investigating novel dinoflagellate parasites in either decapod or other host types, but in such instances these tests should be used in conjunction with PCR and DNA sequencing in order to clearly identify the dinoflagellate taxa present.

The anti-DVNP IFA does not rely on DNA amplification and detects intact cells. In the present study the IFA clearly distinguished *Hematodinium perezi* parasites in haemolymph samples from heavily infected ethanol-preserved *C. sapidus* samples from the USA, demonstrating its utility for the detection of *Hematodinium* in preserved samples. The IFA was able to provide confirmation of a low intensity *Hematodinium* infection in *L. gaimardii* from Port Phillip Bay, and this provided confirmation of live parasites in this host. The IFA was also effective on *Hematodinium* cells derived from *Nephrops norvegicus* (Norway lobster) from Scotland [22]. Given the wide geographic separation between Chesapeake Bay (USA), Scotland (UK) and Port Phillip Bay (southern Australia) and the suggestion that the USA, Europe and Australia likely represent different genotypes or species of *Hematodinium* [3,8], this demonstrates that the DVNP IFA has worldwide utility. Furthermore, the ability to resolve closely spaced nuclei in three dimensions using fluorescence microscopy means that this diagnostic approach can be used on even very densely concentrated cell samples. By contrast, histological interpretation of haemolymph smears requires that cells be well spread out in a single layer and examined carefully at high magnification to allow interpretation of morphological features. Quantitation of parasites to haemocytes can be achieved by counter staining cell nuclei with a fluorescent DNA stain, and this in turn provides further corroboration of the nucleus-localized DVNP labeling. Alternatively, the screen could be used to detect the presence of parasites without quantitative information in cases where cells are too rare to be reliably detected in quantifiable subsamples, as was found in the wild samples from Port Phillip Bay.

Previous IFA diagnostic screens for *Hematodinium* [25,26] relied on antiserum generated against whole *Hematodinium* cells. Although they achieved demonstrable improvements in clarity and sensitivity compared with conventional histological stains, antibodies targeted to whole *Hematodinium* are available only for specific hosts and geographical regions. These antibodies are not reliable for use on different species of *Hematodinium* [17,20]. They are also known to cross-react with other apicomplexan parasites due to shared epitopes [27] and with proteins from culture medium [21]. Small et al. (2007) [28] developed an in-situ hybridization (ISH) technique, which used a digoxigenin-labeled DNA probe to successfully visualize parasites from a variety of hosts in the UK and USA [28]. However, as with PCR, DNA probes need to be carefully validated for each new host and parasite species to ensure that they do not bind to host DNA. By contrast, DVNPs are restricted to dinoflagellates and occur in a known cell localization, the nucleus, providing further corroboration of positive signals [22]. 

Western blot-detection of DVNP provided a further approach to assessing *Hematodinium* infections in decapod crustacean hosts. Owing to the high positive charge of these proteins, a pre-extraction of acid soluble proteins can eliminate much of the non-target protein material from samples, and provide a more sensitive screen for *Hematodinium* parasites. The approach lends itself well to a high throughput sampling strategy, including pooling samples for batch detection, and a level of quantitation of parasite loads.

The advantage of protein-based immunodiagnostic techniques such as Western blot and enzyme-linked immunosorbent assay (ELISA) is that, like PCR, they can by used to concurrently test many samples, which can be advantageous in routine field sampling where large numbers of samples must be collected and processed on a regular basis. The previously developed Western blot detection assay for *Hematodinium* by Stentiford et al. (2001) [21] was assessed to have a sensitivity of 204 parasites ml^-1^ of host haemolymph, however this measure of sensitivity was obtained using a dilution series of heavily infected sample diluted in SDS-sample buffer. In heavy infections of *Hematodinium*, the haemolymph is packed with parasite cells and contains very few haemocytes [1,4]. Thus, the Western blot developed by Stentiford was not fully assessed for cross-reactivity against haemolymph proteins, which are highly abundant in low-level infections and virtually absent from heavy infections. Small et al. (2002) [29] developed an enzyme-linked immunosorbent assay (ELISA) using the same polyclonal antibody to *Hematodinium* cells. The ELISA was assessed as having a sensitivity of 50 parasites ml^-1^ of host haemolymph, again only using H_2_O to dilute heavily infected sample. Thus the DVNP Western blot screen that we have developed is apparently more sensitive than these previously reported approaches and, further, has been tested in more relevant haemolymph sample conditions. 

The size-separation of proteins during the Western blot approach allowed direct analysis of the cross-reactivity of the antibody to DVNP on mixed samples of parasites and host haemolymph and haemocytes. The sensitivity and specificity of the antibody to DVNP indicate that it is a suitable candidate for development of an ELISA using either whole protein or extracts enriched for target protein. Furthermore, owing to the fact that thus far *Hematodinium* is the only known parasitic dinoflagellate of decapod crustaceans, such an ELISA could potentially provide host and region independent detection and quantification of *Hematodinium* in such hosts, and from a large number of field samples. A limitation of detection based on haemolymph is the possibility of missing sessile life stages of the parasite, which are entirely absent from haemolymph or only seldom circulate. However, both the IFA and the Western blot screens could be applied for screening samples of host organs and tissues as well as haemolymph. Importantly, both screens show the potential to overcome problems with the sensitivity and/or adaptability of current methods for the detection of *Hematodinium*, particularly in new decapod host species, new geographical areas, and where very low-intensity infections occur. The IFA was able to clearly visualize parasites in samples of *L. gaimardii* when no obvious signs of infection where detected by inspection of conventional haemolymph smears. Such highly sensitive and versatile methods of detection are needed in the investigation and monitoring of prevalence, distribution, transmission and progression of *Hematodinium* infections in decapod crustacean hosts. 

## Supporting Information

Figure S1 **Custal W alignment of 403 bp, partial 18S rDNA sequence from *Hematodinium* derived from *L. gaimardii*, *O. australiensis* and *P. chabrus* from Port Phillip Bay and *Nephrops norvegicus* [GenBank Accession No. FJ844429].**
(EPS)

Figure S2 **Western blot detection of DVNP.** 20 µg of protein lysate derived from a 3,000 cells ml^-1^ pure *Hematodinium* culture was screened (labeled *Hem*). A reactive band of size consistent with DVNP was detected. Purified recombinant DVNP acted as a control (labeled DVNP).
(EPS)
